# Short- and long-term impact of adapted physical activity and diet counseling during adjuvant breast cancer therapy: the “APAD1” randomized controlled trial

**DOI:** 10.1186/s12885-019-5896-6

**Published:** 2019-07-25

**Authors:** Marion Carayol, Gregory Ninot, Pierre Senesse, Jean-Pierre Bleuse, Sophie Gourgou, Hélène Sancho-Garnier, Chakib Sari, Isabelle Romieu, Gilles Romieu, William Jacot

**Affiliations:** 1 IAPS laboratory “Impact of Physical Activity on Health”, University of Toulon, Avenue de l’Université, 83957 La Garde, France; 2 Val d’Aurelle Montpellier Cancer Institute (ICM), 208, Avenue des Apothicaires, Parc Euromédecine, 34298 Montpellier Cedex 5, France; Montpellier University, 34000 Montpellier, France; 3 Section of Nutrition and Metabolism, International Agency for Research on Cancer, 150 Cours Albert Thomas, 69372 Lyon, Cedex 08 France; 4 Laboratory Epsylon, EA 4556 Dynamics of Human Abilities & Health Behaviors, University of Montpellier, Rue du Pr. Henri Serre, 34000 Montpellier, France; 5 Center for Research on Population Health, National Institute of Public Health, Mexico City, Mexico; 6 Hubert Department of Global Health, Emory University, Atlanta, GA USA; 7 Institut de Recherche en Cancérologie de Montpellier (IRCM), Inserm U1194, Université de Montpellier, Institut du Cancer Montpellier (ICM), Montpellier, France

**Keywords:** Breast cancer, Adjuvant, Exercise, Diet, Intervention, Fatigue, Quality of life, Supportive care

## Abstract

**Background:**

Patients with breast cancer undergoing chemotherapy and radiotherapy experience fatigue and other treatment side effects. Integrative therapies combining physical activity and dietary counseling are recommended; however to date no large randomized controlled trial has been conducted during adjuvant therapy. The Adapted Physical Activity and Diet (APAD) intervention was evaluated for its ability to decrease fatigue (primary outcome), anxiety, depression, body mass index (BMI), and fat mass, and enhance muscular and cognitive performances, and quality-of-life (QoL).

**Methods:**

Women diagnosed with early breast cancer (*N* = 143, mean age = 52 ± 10 years) were randomized to APAD or usual care (UC). APAD included thrice-weekly moderate-intensity mixed aerobic and resistance exercise sessions and 9 dietetic consultations. Patient-reported outcomes (PROs) and anthropometric, muscular, and cognitive variables were measured at baseline, 18 weeks (end of chemotherapy), and 26 weeks (end of radiotherapy and intervention), and at 6- and 12-month post-intervention follow-ups. Multi-adjusted linear mixed-effects models were used to compare groups over time.

**Results:**

Significant beneficial effects of the APAD intervention were observed on all PROs (i.e., fatigue, QoL, anxiety, depression) at 18 and 26 weeks. The significant effect on fatigue and QoL persisted up to 12-month follow-up. Significant decreases in BMI, fat mass, and increased muscle endurance and cognitive flexibility were observed at 26 weeks, but did not persist afterward. Leisure physical activity was enhanced in the APAD group vs UC group at 18 and 26 weeks. No significant effect of the intervention was found on major macronutrients intake.

**Conclusions:**

A combined diet and exercise intervention during chemotherapy and radiotherapy in patients with early breast cancer led to positive changes in a range of psychological, physiological and behavioral outcomes at the end of intervention. A beneficial effect persisted on fatigue and QoL at long term, i.e., 1 year post-intervention. Diet-exercise supportive care should be integrated into the management of early breast cancer patients.

**Trial registration:**

The APAD study was prospectively registered on ClinicalTrials.gov (NCT01495650; date of registration: December 20, 2011).

**Electronic supplementary material:**

The online version of this article (10.1186/s12885-019-5896-6) contains supplementary material, which is available to authorized users.

## Background

With more than 1.6 million new cases in 2012 worldwide, breast cancer (BCa) remains the highest incident cancer in women [1]. In France, it was responsible for 31.2% of all women cancer cases in 2015 which induces a large number of women being treated with adjuvant cancer therapy and then living with its side effects. Indeed, current cancer receiving chemotherapy and/or radiotherapy produce deleterious physiological and psychological effects [2–6] including pain, decreased cardiac function, muscle wasting, weight gain, cancer-related fatigue, and psychological distress.

With a 70–100% prevalence, cancer-related fatigue was reported as the most distressing and common symptom by cancer patients undergoing adjuvant cancer therapy, even more than pain, nausea and vomiting which can generally be managed by medications [2, 7–9]. Cancer-related fatigue has been defined by the National Comprehensive Cancer Network as “a distressing, persistent, subjective sense of physical, emotional and/or cognitive tiredness or exhaustion related to cancer or cancer treatment that is not proportional to recent activity and interferes with usual functioning” [7]. Fatigue commonly occurs within a symptom cluster [3, 5, 6, 10–15] including depression, anxiety, pain, reduced activity level, cognitive functioning impairment, comorbidities, nutritional and anthropometry changes, etc., resulting in impaired quality-of-life (QoL) [4] and affecting cancer prognosis for some of them [16–19]. Exercise and nutrition consultations are two integrative therapy components recommended by the National Comprehensive Cancer Network to relieve side effects, and especially cancer-related fatigue during active treatment [7]. Exercise for cancer patients should associate moderate intensity aerobic exercise and muscle strengthening exercises [20]. Nutrition should be based on the World Cancer Research Fund (WCRF) guidelines after a cancer diagnosis [21] i.e., keep a healthy weight, eat more plant-based foods, limit red and processed meat, limit energy-dense foods, salt, sugary drinks, alcohol, and do not rely on dietary supplements. Exercise may help improve physical fitness, fatigue, QoL, psychological distress and cognitive abilities [22–27], while nutritional consultations may help manage nutritional disorders such as anemia, diarrhea, nausea, and vomiting that contribute to cancer related-fatigue [7, 28, 29]. In addition, the combination of exercise and dietary interventional components has led to significant weight loss in BCa survivors after adjuvant chemotherapy/radiotherapy [30–32]. In patients undergoing adjuvant chemotherapy and/or radiotherapy for BCa, to our knowledge two RCTs assessed exercise and diet in combination, but both were designed as pilot trials with less than 30 patients par randomization group [33, 34]. Therefore, there is a need to evaluate the benefits of an exercise-diet intervention during adjuvant chemotherapy and radiotherapy in a well-powered RCT.

Moreover, most of the RCTs that assessed exercise and/or diet interventions during chemotherapy/radiotherapy had a relatively short-term follow-up (no more than 6 months [34–40]). In their recent meta-analysis, Furmaniak et al. [23] concluded that longer-term evaluation is required due to long-term side effects of adjuvant treatment.

The Adapted Physical Activity and Diet counseling (APAD) trial was designed to assess the 1-year follow-up effects of an exercise-diet intervention delivered during a 6-cycle adjuvant chemotherapy regimen followed by radiotherapy in patients with early BCa. We hypothesized that APAD would yield beneficial effects as compared to usual care (UC) on cancer related-fatigue as a primary outcome. Secondary outcomes of our study include QoL, anxiety and depressive symptoms, body mass index (BMI), body composition, cognitive and muscular functions, chemotherapy adherence, physical activity practice, and nutritional intake.

## Methods

The protocol of the APAD1 study has been described previously [41]. The APAD1 study has been designed and implemented in order to evaluate the impact of a exercise and nutrition-based supportive care intervention named APAD during chemotherapy and radiotherapy in BCa patients. The APAD1 study was located in a cancer health center from south of France (the Montpellier Cancer Institute). The APAD intervention was evaluated as compared to UC that did not involve specific exercise or nutrition care for BCa patients.

### Experimental design and population study

The APAD1 study was a two-armed, randomized, controlled, prospective trial (see Aditional file 1: Figure S1). Eligible participants were women aged 18–75 years with histological proven and newly (less than 6 months) diagnosed non-metastatic BCa, whatever their baseline physical activity level or dietary intakes. The patients were enrolled after undergoing curative surgery. All patients were planned for 6 cycles of adjuvant chemotherapy (including epirubicin / cyclophosphamide / 5-fluorouracil for 3 cycles every 3 weeks [FEC100 protocol], followed by docetaxel for 3 cycles every 3 weeks), followed by 6 weeks of radiotherapy administered at the Montpellier Cancer Institute [ICM, Montpellier, France]) [42]. All participants were recruited at the ICM. Exclusion criteria were medical contra-indications to moderate intensity physical activity, inability to attend intervention sessions or assessments, and a difficulty or disability preventing the patient from correctly understanding the trial information or requirement. The study received agreement from ethical and institutional review boards and was prospectively registered on ClinicalTrials.gov (NCT01495650; date of registration: December 20, 2011).

### Procedure

Before chemotherapy, potential participants were identified by the hospital medical oncologists with help from clinical research assistants. All of the patients that were proposed to be part of the APAD1 study were informed about the objective of the study and the potential benefits of diet and exercise on fatigue during adjuvant therapy. The patients who provided written informed consent and completed baseline assessments were then randomly assigned in a 1:1 ratio to the APAD experimental arm or to the UC control arm (Additional file 1: Figure S1) using a computer-generated program. Block randomization (block size = 4) was performed at the ICM Biometric Unit by using the Stata software version 12 (StatCorp, LLC, College Station, TX, USA). The allocation assignment was concealed from the project directors. Participants, interventionists, and assessors were not masked to group assignment.

### Interventions

#### Overview

The APAD program was implemented during chemotherapy and radiotherapy (approximately 26 weeks) and included: *(i)* exercise sessions planned thrice-weekly including individually supervised hospital-based exercise sessions and non-supervised home-based sessions, combining one muscle strength session and two aerobic sessions each week. Hospital-based supervised exercise sessions were planned every 3 weeks on the same days of chemotherapy and radiotherapy to avoid any additional cost; the program included 9 supervised sessions at the ICM hospital center and all other sessions were home-based unsupervised sessions. *(ii)* Nine hospital-based and face-to-face nutritional therapeutic education sessions targeting body weight control and to modify feeding behaviors according to the WCRF recommendations [21]. The nutritional sessions were planned on the same days as supervised hospital-based exercise sessions. For the participants who met exercise and/or dietary intakes guidelines at baseline, the intervention was tailored as they had to maintain their exercise level and their dietary intakes in accordance with the guidelines all along the intervention period. The content of the APAD intervention has been previously described [41].

#### Exercise

The exercise program was delivered by exercise specialists who are certified (or is eligible to certification) in adapted physical activity by the French University of Sports Sciences and combined aerobic and muscle strengthening exercises in compliance with the recommendations established for cancer patients [20, 43]. About the exercise dose, guidelines of 150 min of moderate to vigorous physical activity (MVPA) per week associated with strength training have been advanced for cancer patients whatever cancer site or treatment period [20, 44]. Exercising while receiving chemotherapy or radiotherapy involves specific brakes, especially the loss of physical condition induced by chemotherapy in BCa patients is an important brake in doing relatively high doses of MVPA, as compared to exercising after adjuvant therapy as a cancer survivor. In a meta-analysis, we have investigated the optimal dose of exercise to be prescribed to BCa patients undergoing chemotherapy and/or radiotherapy [22]. Inverse dose–response relationships were observed for fatigue and QoL, supporting the hypothesis of greater improvements of fatigue and QoL with lower weekly prescribed exercise doses (< 12 Metabolic Equivalent of Task (MET).h/week). These findings generated the hypothesis that the prescription of a program targeting 8–10 MET h/week could be well adapted for patients with BCa receiving adjuvant therapy [22]. An adapted program then could consist in one resistance session for principal muscular groups and two moderate intensity 30 to 45 min aerobic sessions per week. This is the prescribed dose that was tested in the APAD1 study.

Exercise sessions were planned thrice-weekly including individually supervised hospital-based exercise sessions and non-supervised home-based sessions, combining one muscle strength session and two aerobic sessions each week. Every session starts with warm-up for 10 min including joint rotations (fingers, wrists, elbows, shoulders, neck, hips, knees, ankles, feet and toes) for 3 min, slow jogging for 5 min, and cross-over stepping, thighs lift and heels to buttocks for 2 min. Every session ends with cool-down followed by flexibility exercises (10 min).

Strength sessions (once a week) targeted 6 main muscle groups (hamstrings, quadriceps, buttocks, abdominal, back, shoulders/arms) by asking patients to achieve 6 different tasks. Each task was performed for 2 to 5 sets with 6 to 12 repetitions. Two to 5 different tasks with increasing difficulty were available for each muscle group. Every 6 weeks the exercise specialist proposed a 2-repetition or 1-set increase, and/or shift for more difficult task according to patient’s physical condition and progression.

Aerobic exercise sessions were performed at moderate intensity and adapted to patient’s physical condition and progression in the range of 50–75% of the maximum heart rate for 30 to 45 min. Initial exercise intensity was individualized but generally began at 50 to 55% of the maximum heart rate and progressed to 65 to 75% of the maximum heart rate by weeks 20 to 26. Initial exercise duration was also individualized but generally began between 25 to 35 min per session and achieved 40 to 50 min per session by weeks 20 to 26. The starting point for exercise prescription was determined according to the maximum heart rate estimated as 220 – age of the patient [45], and patient exercise history and current practice. Rate of progression was individualized according to the severity of treatment-related side effects. When patients presented health troubles, important fatigue, or any symptom that could limit exercise, an adapted decreased dose was proposed to patients by the exercise specialist. Supervised hospital-based sessions were achieved on a cycloergometer. For home-based practice, patients were proposed various modalities of aerobic exercise (e.g., walking, jogging, cycling, dancing/fitness, swimming) to aid compliance to the program and promote enjoyment [46].

Hospital-based supervised exercise sessions aimed to provide the patients with relevant instructions to allow reproducibility at home and increase autonomy. Every supervised session was based on theoretically grounded specific behavioral targets (e.g., problem-solving barriers, self-efficacy, social support) [47] and behavior change techniques (e.g., provide information about behavior-health link and consequences of practice, provide instruction and demonstrate the behavior, set graded tasks, prompt self-monitoring of behavior, plan social support or social change) [48] (see Additional file 2: Table S1) [41] to improve behavioral change and patient’s adherence. Hospital-based supervised exercise sessions were scheduled on the same day as chemotherapy administration and during radiotherapy, at the frequency of one every 3 weeks. In total, 9 hospital-based supervised exercise sessions were planned in the course of the intervention. Each session lasted approximately 50 to 70 min.

Home-based non-supervised sessions were planned 3 times per week, except that only 2 home-based sessions a week were scheduled on the weeks involving one supervised hospital-based exercise session. Precise written instructions for home-based sessions were given to patients in the educational and personable APAD-Moving workbook (French language, available on request) including information about disease and reasons for being physically active during cancer active treatment, written instructions illustrated with pictures for performing home exercises (e.g.*,* warm-up content, muscle tasks, prescribed number of series/repetitions with space for potential incrementation, aerobic intensity and duration, flexibility tasks), schedule for planned home-based sessions, and patient log to evaluate patient adherence. The strength-based exercise that were taught to participants during the supervised sessions did not need any particular material so that they could easily be done at home. Patients were asked to fill in the adherence log at home with whether planned sessions were achieved or not, number of achieved muscular exercises, duration of session, rating of perceived exertion (on scale of 1 to 10), reason for missed session and, any commentary that they would like to discuss with the exercise specialist at the next supervised session. The APAD-Moving workbook included a range of behavior changes techniques [48] (e.g., provide information about behavior-health link and consequences of practice, provide instruction and demonstrate the behavior, set graded tasks, prompt self-monitoring of behavior).

#### Diet counseling

APAD patients received 9 face-to-face individual sessions of diet counselling from a dietician who is certified (or is eligible to certification) by a French University. Each session lasted approximately 30 min.

In the course of chemotherapy, 6 diet sessions were scheduled to reach balanced dietary intakes advising patients for controlling weight and for managing with chemotherapy potential toxicities and side-effects. Then 3 more sessions planned in the course of radiotherapy for all APAD patients: in patients with BMI < 30, weight control was pursued; in patients with BMI ≥ 30, weight normalization was targeted (i.e., decreasing BMI below to 30 by the end of adjuvant therapy).

Each consultation involved *(i)* nutritional status evaluation, *(ii)* nutrition care tailored to the patient’s caloric needs and potential toxicities related to treatment and, *(iii)* nutritional education.

##### Nutritional status evaluation

Nutritional status was evaluated based on the usual weight, the current weight and the weight measured one to six months prior to study enrolment according to the French National Authority for Health criteria. The dietician assessed the patient’s daily energy requirement by computing the basal metabolic rate (BMR) [49]; the BMR was multiplied by 1.3 to allow for energy needed for daily living activities, and then in APAD patients only the BMR was multiplied by 1.2 to allow for energy needed for exercise.

##### Nutrition care

Nutrition care aimed at weight control through balanced dietary intakes tailored to the patient’s energy intake needs and potential toxicities related to treatment. At each session, the dietician evaluated dietary intakes with 24 h-recall and appetite using a 10-point visual analogue scale. Calories and nutrients were computed by entering food intakes on a nutritional analysis software [50]. Then the dietician verified the patient’s intakes to be in line with the following guidelines: *(i)* daily energy intake was compared to the estimated daily energy needs, *(ii)* patients were guided to regularly distribute their dietary intakes as 3 main meals with an optional snack in the afternoon, *(iii)* macronutrient distribution was compared to the French dietary reference intakes for balanced diet (i.e., 30–35% of lipids, 50–55% of carbohydrates, and 10–15% of proteins) [35] and, *(iv)* food groups intake were guided to meet the recommendations of the WCRF [21]. If either patient’s habits did not correspond to these guidelines, or daily energy intake was higher or lower than 10% of the estimated daily energy needs, the dietician counselled modifications regarding foods, nutrients, meals, and calories distribution. When patient’s BMI was higher than 30 by the end of chemotherapy, a new weight goal was settle by decreasing the patient’s BMI within the range of 25 to 30. A new range of daily energy need was then estimated with a corresponding distribution according to foods groups balance and WCRF guidelines [21]. Patients were given a printed example of food groups, servings and distribution they may eat on a typical day. In the following sessions, the dietician computed again patient’s intakes and adapted her advice according to the evolution of patient’s intakes. Specific advice was given to patients for the management of chemotherapy potential toxicities and side-effects [51]. In case of nausea or vomiting, patients were recommended to eat foods at room or cold temperature, to avoid sugary, fatty, and highly flavored foods, to eat steamed vegetables, dry foods (e.g., toasted or crisp bread), fresh foods (e.g., yogurts, fresh fruits, sorbet), to take cool drinks outside meals, to eliminate exciting drinks (e.g., tea, coffee, alcohol). In case of mucositis, patients were advised to avoid acid and spicy foods (e.g., lemon, mustard, vinegar), to keep their month wet by drinking frequently, to eat smooth and cooked foods at room temperature. Patients were recommended to: increase or diminish dishes seasoning (e.g., spices, herbs, salt, sugar) according to patient’s tasting in case of taste disorders; increase starchy foods (e.g., potatoes, rice, pasta), béchamel sauce with green vegetables, and white meat, eggs or fish rather than red meat; and to start meals by taking acid beverages or fruits (e.g., grapefruit, pineapple) in case of metallic taste.

##### Nutritional education

Nutritional education aimed to teach the patients with the principles of a well-balanced and healthy diet based on WCRF guidelines [21], inform about industrial food products packaging, and fight against preconceived ideas, by using practical applications and educational games. Each session was based on specific education targets (Additional file 3: Table S2). Nutritional education was tailored to patients’ habits and means, precariousness level, and cultural and social environment.

#### Missed sessions

##### During chemotherapy

Regarding hospital-based supervised sessions, missed supervised exercise or diet counseling sessions were not possible to be rescheduled as patients only come to the hospital once every three weeks during chemotherapy. In case of missed supervised session, a phone call was made to the patient by the exercise and/or dietician specialists. Discussion focused on reasons for not attending the session, patient adherence in the last three weeks, encourage the patient attending future exercise or diet counseling sessions taking into account its difficulties and deliver education targets and content (if possible) of the missed session.

##### During radiotherapy

As most of the patients come to the hospital every weekday during radiation therapy, supervised hospital-based missed sessions were rescheduled as soon as possible.

### Control

The control group was a UC group without any diet or exercise intervention. There was no particular material delivered period to the control group during the intervention. The UC patients were not asked to limit exercise practice or eat/avoid specific foods during the intervention period.

### Outcome measures

Five repeated assessments were conducted at the ICM. These occurred at baseline, just before the start of adjuvant chemotherapy (T0); the end of chemotherapy (T1); end of radiotherapy; immediately post-intervention (T2); 6-month follow-up (T3); and 1-year follow-up (T4) (Additional file 1: Figure S1).

Details about the assessment planning and tools employed were published previously [41]. Subjective patient-reported outcomes (PROs) were evaluated at all assessment times. Cancer-related fatigue (primary outcome) was assessed using the Multidimensional Fatigue Inventory (MFI) [52]. QoL was assessed by using the EORTC QLQ-C30 [53]. Anxiety and depression symptomatology was evaluated using the Hospital Anxiety Depression Scale [54]. All questionnaires were available and validated in French. Objective outcomes were mostly assessed at T0, T2, T3, and T4. Power and strength of 10 successive vertical jumps allowing a 10-s interval were measured by the Myotest® accelerometer system [55]. The power/strength maintained during the task was estimated by the ratio of the last two to the first two jumps that was considered as an indicator of muscular fatigue in healthy individuals and athletes [56–58]. Lower limb muscle endurance was measured at 15 s and 30s using the 30-s chair stand test [59]; the ratio of 15–30s to 0–15 s number of repetitions was computed to assess an index of lower limb muscle fatigability. Attentional capabilities (i.e.*,* alertness, working memory and flexibility) were assessed using the Test of Attentional Performance (TAP software, version 2.3) [60]. Physical activity during the past week was collected by face-to-face interviews using the Global Physical Activity Questionnaire (GPAQ) [61] at all of the assessment times. The SenseWear Pro Armband™ (version 3.0) [62] accelerometer data were collected at T0 and T2 and were included in the analysis if patients provided at least 3 days of wear for ≥ 10 h/day. Several variables were considered in the analysis: total duration of physical activity (min/day), total duration of moderate physical activity (min/day), average METs (METs.h/day) and, sedentary time (min/day). Moderate intensity physical activity corresponded to measured intensity from 3 to 6 METs and sedentary time corresponded to less than 3 METs.

Anthropometry measures included weight, height, and BMI at all of the assessment times, and body composition at T0, T2, T3, and T4 by using Bioelectrical Impedance Analysis [63]. Dietary intakes were measured at T0, T2, and T4 by asking patients to complete a 3-day food record [64], with the foods and beverages consumed for 3 consecutive days (of which one weekend day), and were entered into nutritional analysis software to compute calories and nutrient intake [50]. Chemotherapy observance was recorded in order to calculate the relative dose-intensity (RDI) as the ratio of delivered dose intensity of chemotherapy to standard dose intensity.

### Statistical analysis

A two-point reference for the general fatigue MFI sub-scale was established as a valuable minimal clinically important difference in cancer patients with active treatment by Purcell et al. [65]. With 70 participants per group, our trial had 90% power to detect a between-group difference of 2 points (standard deviation [SD] = 4 [65, 66]), which corresponded to a 0.5 effect size, on the general fatigue scale of the MFI [52] at each time point (T1, T2, T3, T4), considering the general fatigue mean level observed in the APAD group at baseline (Table 2), and allowing for a repeated measures design [67] and a 10% loss-to-follow-up. A two-tailed *P* value was set at .05.

Linear mixed models were used to model each outcome measure over post-randomization time points (maximum of 4) and to compare the mean differences between groups across time based on the interaction effect between time (T1, T2, T3 and T4) and group (APAD, UC). An unstructured covariance matrix for residuals was assumed. Participants were considered as random effects and group, time, and arm-by-time interaction as fixed effects. Models were adjusted for the baseline value of the outcome, age, and surgery type. First, analyses were conducted following the intention-to-treat principle, using all available data without any missing data imputation.

Because of a drop-out difference in APAD vs Control, the risk of selection bias was explored by comparing baseline characteristics (i.e.*,* sociodemographic, clinical, PRO, muscular, anthropometry, and behavioral variables) of the included patients with complete data (i.e.*,* the primary outcome was collected at all of the assessment times) and incomplete data (i.e.*,* the primary outcome was missing at one [or more than one] of the assessment times).

All statistical tests were two-sided, with a 5% level of significance. Effect size of 0.2 should be considered as a ‘small’ effect size, 0.5 represents a ‘medium’ effect size and 0.8 a ‘large’ effect size. Statistical analyses were conducted in Stata version 12 (StatCorp, LLC, College Station, TX, USA).

## Results

**Fig. 1:**
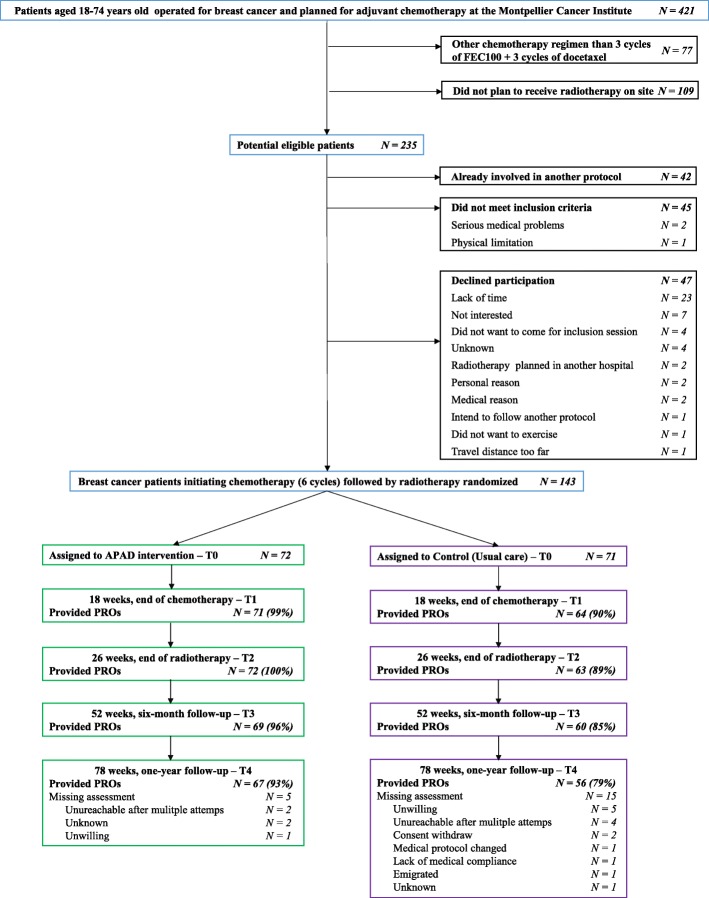
CONSORT flow diagram of patients with breast cancer participating in the APAD1 study. Note: The primary outcome (assessed by the Multidimensional Fatigue Inventory) is part of the Patient-Reported Outcomes (PROs)

From December 2010 to April 2013, 143 patients out of 235 (61%) eligible patients were randomized to APAD (*N* = 72) or UC (*N* = 71) and followed during 1.5 year (Fig. 1). PRO data were collected from 94, 94, 90, and 86% of patients at T1, T2, T3, and T4, respectively. Baseline characteristics of the patients are presented in Table 1 and include women with a median age of 52 years and a median BMI of 25.5 kg/m^2^. On average, APAD participants attended 67% of the planned exercise sessions (*n* = 48 of 72), 67% of the supervised sessions (*n* = 6 of 9), and 67% of the home-based sessions (*n* = 42 of 63). On average, 71% of the aerobic exercise sessions (*n* = 34 of 48) with 41 ± 25 min duration and 58% of the resistance exercise sessions (*n* = 14 of 24) were completed. The number of exercise sessions achieved each week by the APAD patients are presented on the Additional file 4: Figure S2. A large majority of the APAD patients have completed at least one exercise session every week of the program (from 81% on week 18 to 98% on week 1), and in general a majority of the APAD patients have completed 3 or more exercise sessions each week on the program (from 44% on week 11 to 80% on week 3). Reasons for not achieving exercise planned sessions reported by patients are displayed on Additional file 5: Figure S3. The stated reasons from the most to the less frequent were fatigue (33%), lack of motivation (24%), pain (13%), health problem (9%), nausea (6%), professional or familial imperative (5%), refusal (2%), bad weather (2%), and other (6%). Regarding dietary counseling, APAD participants attended 97% of the planned consultations (on average, *n* = 9 of 9). No serious adverse events were related to exercise or diet intervention.

**Table 1:** Characteristics of included patients with breast cancer

	Total (*N* = 143)		UC (*N* = 71)		APAD (*N* = 72)	
Age, *mean (sd)*	51.6	*(10.1)*	52.1	*(9.3)*	51.2	*(10.9)*
Weight, *mean (sd)*	68.5	*(13.9)*	69.6	*(14.1)*	67.5	*(13.7)*
BMI, *mean (sd)*	25.5	*(5.3)*	25.8	*(5.3)*	25.2	*(5.4)*
Obese, *N (%)*	26	*(18.2)*	15	*(21.1)*	11	*(15.3)*
Post-menopausal, *N (%)*	67	*(46.8)*	37	*(52.1)*	30	*(41.7)*
Tobacco smoking, *N (%)*						
Non-smoker	65	*(45.4)*	30	*(42.2)*	35	*(48.6)*
Smoker	36	*(25.2)*	22	*(31.0)*	14	*(19.4)*
Former smoker	42	*(29.4)*	19	*(26.8)*	23	*(31.9)*
Marital status, *N (%)*						
Single/divorced/widowed, no child	3	*(2.1)*	2	*(2.8)*	1	*(1.4)*
Single/divorced/widowed, with child	15	*(10.5)*	7	*(9.9)*	8	*(5.6)*
Married/Living together, no child	7	*(4.9)*	3	*(4.2)*	4	*(2.8)*
Married/Living together, with child	118	*(82.5)*	59	*(83.1)*	59	*(81.9)*
Education level, *N (%)*						
No qualifications	23	*(16.1)*	14	*(19.7)*	9	*(12.5)*
Secondary level	29	*(20.3)*	15	*(21.1)*	14	*(19.4)*
Completed high school	31	*(21.7)*	11	*(15.5)*	20	*(27.8)*
Completed ≥2 years at University	60	*(42.0)*	31	*(43.7)*	29	*(40.3)*
Usual professional status, *N (%)*						
Full or part-time employed	95	*(66.4)*	43	*(60.5)*	52	*(72.2)*
Retired	36	*(25.2)*	19	*(26.8)*	17	*(23.6)*
Unemployed/Medical leave	12	*(8.4)*	9	*(12.7)*	3	*(4.2)*
Surgery type, *N (%)*						
Lumpectomy	60	*(42.0)*	26	*(36.6)*	34	*(47.2)*
Quadrantectomy	57	*(39.9)*	37	*(52.1)*	20	*(27.8)*
Mastectomy	26	*(18.2)*	8	*(11.3)*	18	*(25.0)*
Cancer stage^a^, *N (%)*						
Stage I	62	*(43.3)*	33	*(46.5)*	29	*(40.3)*
Stage IIa/IIb	62	*(43.3)*	29	*(40.8)*	33	*(45.8)*
Stage IIIa/IIIc	18	*(12.6)*	9	*(12.7)*	9	*(12.5)*
Breast cancer subtype, *N (%)*						
Triple negative	32	*(22.4)*	13	*(18.3)*	19	*(26.4)*
HER2+, ER+, and/or PR+	17	*(11.9)*	9	*(12.7)*	8	*(11.1)*
HER2+, ER−, and PR−	1	*(0.7)*	1	*(1.4)*	0	*(0.0)*
HER2-, ER+, and/or PR+	93	*(65.0)*	48	*(67.6)*	45	*(62.5)*

*Abbreviations*: *APAD* Adapted Physical Activity and Diet counseling intervention, *UC* Usual Care

^a^One APAD participant with pTX (i.e., adenocarcinoma in an ectopic axillary breast tissue)

**Fig. 2:**
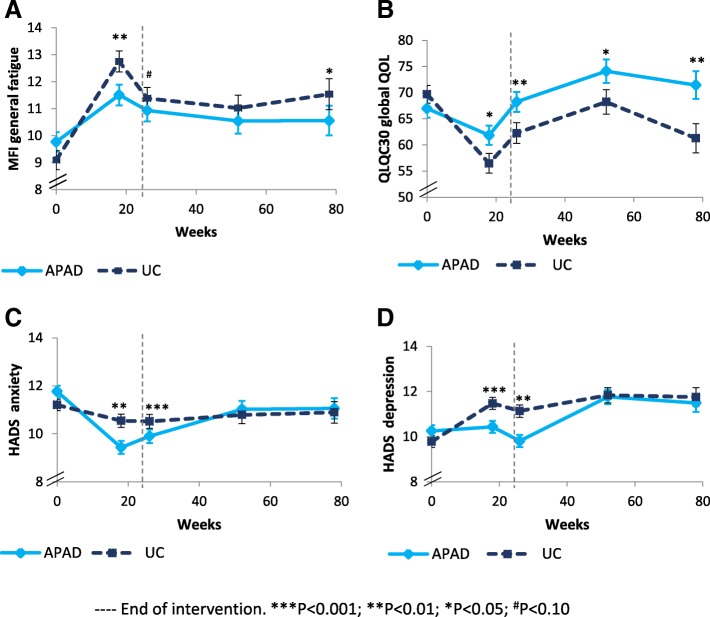
Effects of the APAD intervention on (**a**) fatigue, (**b**) QoL, (**c**) anxiety, and (**d**) depression in women with breast cancer at the end of chemotherapy, end of radiotherapy, and the 6-month and 1-year post-intervention follow-ups after the end of radiotherapy. Note: Means and standard errors are estimated from adjusted linear mixed analyses. Baseline score is the adjusted score. APAD: Adapted Physical Activity and Diet counseling intervention; UC: Usual Care

**Fig. 3:**
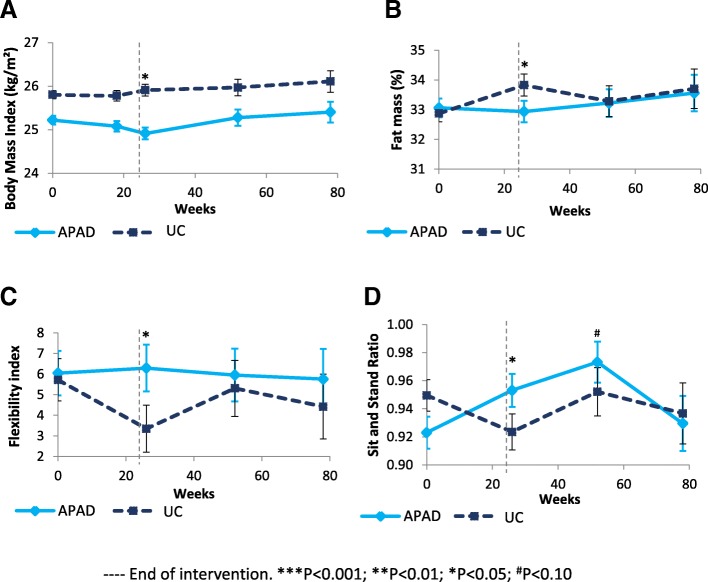
Effects of the APAD intervention on (**a**) BMI, (**b**) fat mass, (**c**) cognitive flexibility, and (**d**) sit-and-stand ratio in women with breast cancer at the end of chemotherapy, end of radiotherapy, and at the 6-month and 1-year post-intervention follow-ups after the end of radiotherapy. Note: Means and standard errors are estimated from adjusted linear mixed analyses. Baseline score is the adjusted score. APAD: Adapted Physical Activity and Diet counseling intervention; UC: Usual Care

Tables 2, 3 and 4, and Figs. 2 and 3 present data and outcome changes due to the intervention.

**Table 2:** Mean values at baseline, and between-group differences and at the end of chemotherapy, the end of radiotherapy and at 6-month and 1-year follow-up for PROs

		T0 Baseline	T1–18 weeks Mid-intervention			T2–26 weeks End of intervention			T3–52 weeks 6-month follow-up			T4–78 weeks 1-year follow-up		
		*Mean (SD)*	AMD (95% CI)*	*ES*	*P†*	AMD (95% CI)*	*ES*	*P†*	AMD (95% CI)*	*ES*	*P†*	AMD (95% CI)*	*ES*	*P†*
Fatigue (MFI)														
General fatigue	*UC*	9.2 (3.4)	**−1.72 (−2.72; − 0.71)**	**−0.28**	***0.002***	− 0.92 (− 1.98; 0.13)	− 0.14	*0.081*	− 0.95 (−2.22; 0.31)	− 0.12	*0.103*	**−1.46 (− 2.96; 0.05)**	**− 0.16**	***0.038***
	*APAD*	9.9 (3.7)												
Physical fatigue	*UC*	9.2 (4.2)	**−2.08 (−3.11; − 1.04)**	**− 0.33**	***0.001***	**−1.96 (−3.03; − 0.88)**	**− 0.30**	***0.002***	−0.78 (−2.02; 0.46)	−0.10	*0.207*	**−1.54 (−2.96; −0.11)**	**−0.18**	***0.034***
	*APAD*	9.5 (4.2)												
Mental fatigue^‡^	*UC*	7.6 (3.4)	**−0.15 (− 0.26; − 0.04)**	**− 0.22**	***0.015***	**− 0.18 (− 0.3; − 0.06)**	**−0.25**	***0.004***	**−0.15 (− 0.3; − 0.01)**	**−0.17**	***0.031***	−0.09 (− 0.27; 0.09)	− 0.08	*0.244*
	*APAD*	8.1 (3.7)												
Reduced activities^‡^	*UC*	9 (3.8)	**−0.2 (− 0.31; − 0.09)**	**− 0.30**	***0.002***	**− 0.17 (− 0.29; − 0.06)**	**− 0.25**	***0.010***	**− 0.21 (− 0.34; − 0.07)**	**−0.25**	***0.005***	**−0.17 (− 0.33; 0)**	**− 0.17**	***0.044***
	*APAD*	9.1 (3.9)												
Reduced motivation^‡^	*UC*	6.7 (2.6)	**−0.18 (− 0.29; − 0.07)**	**− 0.28**	***0.007***	**− 0.15 (− 0.26; − 0.04)**	**− 0.22**	***0.026***	− 0.09 (− 0.22; 0.04)	− 0.11	*0.203*	− 0.1 (− 0.26; 0.05)	− 0.11	*0.196*
	*APAD*	6.9 (3.4)												
Quality-of-life (EORTC QLQ C30)														
Global QoL	*UC*	69.5 (18.9)	**7.08 (2.22; 11.95)**	**0.24**	***0.012***	**7.69 (2.6; 12.78)**	**0.25**	***0.008***	**7.58 (1.46; 13.69)**	**0.20**	***0.018***	**11.87 (4.52; 19.21)**	**0.26**	***0.002***
	*APAD*	66.9 (18)												
Physical function	*UC*	88.8 (11.1)	**8.54 (4.56; 12.5)**	**0.35**	***0.000***	**7.44 (3.34; 11.5)**	**0.30**	***0.001***	2.84 (−2; 7.69)	0.10	*0.199*	**8.69 (2.98; 14.4)**	**0.25**	***0.002***
	*APAD*	86.5 (13.7)												
Role function	*UC*	84.5 (23.1)	**13.5 (8.26; 18.6)**	**0.42**	***0.000***	**9.27 (3.83; 14.7)**	**0.28**	***0.003***	3.98 (−2.48; 10.45)	0.10	*0.198*	**8.61 (0.96; 16.3)**	**0.18**	***0.022***
	*APAD*	82.9 (18.9)												
Emotional function	*UC*	66.3 (21.7)	**9.38 (3.5; 15.3)**	**0.26**	***0.014***	2.88 (−3.3; 9.05)	0.08	*0.472*	1.73 (−5.5; 8.97)	0.04	*0.695*	6.4 (−2.08; 14.9)	0.12	*0.172*
	*APAD*	66.5 (19)												
Cognitive function	*UC*	85.5 (17.9)	**8.48 (3.13; 13.8)**	**0.26**	***0.008***	3.87 (−1.7; 9.45)	0.11	*0.219*	2.26 (−4.18; 8.7)	0.06	*0.486*	5.64 (− 1.78; 13.1)	0.12	*0.129*
	*APAD*	85.9 (18.6)												
Social function	*UC*	82.1 (23.3)	6.22 (−0.13; 12.58)	0.16	*0.234*	7.04 (0.45; 13.62)	0.18	*0.174*	0.77 (−7.03; 8.56)	0.02	*0.946*	**13.0 (3.80; 22.3)**	**0.23**	***0.025***
	*APAD*	84.7 (22.5)												
Anxiety and depression (HADS)														
Anxiety	*UC*	11.3 (3.3)	**−1.57 (−2.28; − 0.87)**	**− 0.36**	***0.000***	**−1.08 (− 1.83; − 0.33)**	**− 0.24**	***0.009***	− 0.23 (− 1.18; 0.72)	− 0.04	*0.533*	− 0.3 (− 1.48; 0.88)	− 0.04	*0.515*
	*APAD*	11.9 (3.2)												
Depression	*UC*	9.7 (3.3)	**−1.36 (−2.05; −0.68)**	**−0.32**	***0.001***	**−1.66 (−2.37; −0.95)**	**−0.38**	***0.000***	−0.4 (−1.28; 0.47)	−0.08	*0.305*	−0.59 (−1.69; 0.5)	−0.09	*0.243*
	*APAD*	10.1 (3.4)												

*Abbreviations*: *AMD* Adjusted Mean Differences, *EF* Effect Size of difference between groups, *CI* Confidence Interval, *MFI* Multidimensional Fatigue Inventory, *HADS* Hospital Anxiety Depression Scale, *APAD* Adapted Physical Activity and Diet counseling intervention, *UC* Usual Care

NOTE: Bold font indicates significant difference

^*^Baseline value, surgery type and age-adjusted mean differences and their associated 95% confidence interval estimated with linear mixed model

^†^*P*-value for time*arm interaction estimated with linear mixed model

^‡^Log transformed variables: Mental fatigue – MFI, Reduced activities – MFI, Reduced Motivation – MFI

**Table 3:** Mean values at baseline, and between-group differences and at the end of chemotherapy, the end of radiotherapy and at 6-month and 1-year follow-up for muscular, body composition and attentional outcomes

		T0 Baseline	T1–18 weeks Mid-intervention			T2–26 weeks End of intervention			T3–52 weeks 6-month follow-up			T4–78 weeks 1-year follow-up		
		*Mean (SD)*	AMD (95% CI)*	*EF*	*P*†	AMD (95% CI)*	*EF*	*P*†	AMD (95% CI)*	*EF*	*P*†	AMD (95% CI)*	*EF*	*P*†
Muscular tests														
Sit-and-stand 30s^‡^	*UC*	16.4 (4.9)	–	–	–	**0.07 (0.02; 0.12)**	**0.25**	***0.005***	0.04 (−0.03; 0.11)	0.09	*0.203*	0.07 (−0.03; 0.17)	0.12	*0.125*
	*APAD*	15.3 (3.7)												
Sit-and-stand ratio	*UC*	0.95 (0.12)	–	–	–	**0.04 (0.01; 0.08)**	**0.22**	***0.013***	0.036 (−0.007; 0.078)	0.14	*0.081*	0.01 (− 0.05; 0.06)	0.02	*0.563*
	*APAD*	0.92 (0.11)												
Power ratio	*UC*	0.94 (0.14)	–	–	–	−0.01 (− 0.06; 0.04)	0.09	*0.541*	**−0.07 (− 0.13; − 0.01)**	**−0.19**	***0.041***	−0.02 (− 0.1; 0.06)	− 0.05	*0.508*
	*APAD*	0.96 (0.15)												
Force ratio	*UC*	0.99 (0.14)	–	–	–	0.00 (−0.04; 0.04)	0.01	*0.978*	−0.02 (− 0.07; 0.04)	− 0.05	*0.529*	−0.04 (− 0.11; 0.03)	− 0.09	*0.313*
	*APAD*	1.00 (0.15)												
Attentional performances (TAP)														
Alertness index	*UC*	−0.002 (0.099)	–	–	–	− 0.01 (− 0.04; 0.01)	−0.09	*0.359*	0.00 (−0.03; 0.04)	0.02	*0.918*	0.01 (−0.04; 0.06)	0.04	*0.700*
	*APAD*	0.010 (0.069)												
Flexibility index	*UC*	6.7 (9.4)	–	–	–	**2.58 (0.54; 4.62)**	**0.21**	***0.044***	0.281 (−2.46; 3.03)	0.02	*0.841*	0.972 (−2.49; 4.44)	0.05	*0.594*
	*APAD*	7.2 (9)												
Flexibility: errors^‡^	*UC*	2 (4.1)	–	–	–	**−0.29 (−0.48; − 0.09)**	**− 0.24**	***0.024***	−0.05 (− 0.32; 0.22)	− 0.03	*0.741*	−0.023 (− 0.38; 0.34)	− 0.01	*0.898*
	*APAD*	1.8 (3.4)												
Working memory: errors - omissions^‡^	*UC*	5.9 (6.5)	–	–	–	−0.06 (−0.26; 0.14)	−0.05	*0.915*	0.14 (−0.16; 0.43)	0.08	*0.217*	−0.113 (− 0.51; 0.28)	− 0.05	*0.854*
	*APAD*	3.7 (4.1)												
Anthropometry and body composition														
Weight (kg)	*UC*	69.7 (14.1)	−0.44 (−1.27; 0.39)	−0.09	*0.576*	−1.19 (−2.13; −0.25)	−0.21	*0.051*	−0.45 (−1.8; 0.91)	−0.05	*0.680*	−0.52 (−2.33; 1.29)	−0.05	*0.690*
	*APAD*	67.5 (13.8)												
BMI (kg/m^2^)	*UC*	25.8 (5.3)	−0.17 (−0.48; 0.15)	−0.09	*0.515*	**−0.46 (− 0.82; − 0.11)**	**−0.21**	***0.033***	−0.16 (− 0.66; 0.34)	− 0.05	*0.664*	−0.17 (− 0.84; 0.49)	− 0.04	*0.706*
	*APAD*	25.3 (5.4)												
FM (%)	*UC*	32.9 (7.7)	–	–	–	**−1.10 (−1.97; − 0.22)**	**−0.21**	***0.041***	− 0.27 (− 1.53; 1.00)	− 0.03	*0.717*	− 0.35 (−2.05; 1.35)	− 0.03	*0.708*
	*APAD*	33.1 (7.7)												
MM (kg)	*UC*	45.4 (5.4)	–	–	–	−0.16 (−0.65; 0.33)	−0.05	*0.741*	0.33 (−0.36; 1.02)	0.08	*0.279*	0.56 (−0.34; 1.47)	0.10	*0.174*
	*APAD*	44.2 (4.9)												
MM to FM ratio^§^	*UC*	0.5 (0.2)	–	–	–	0.03 (0; 0.07)	0.17	*0.093*	0.02 (−0.03; 0.07)	0.06	*0.469*	0.01 (− 0.06; 0.08)	0.02	*0.836*
	*APAD*	0.5 (0.2)												
FM - trunk (kg)	*UC*	12.2 (5.6)	–	–	–	**−0.88 (−1.49; − 0.27)**	**− 0.24**	***0.027***	−0.02 (− 0.85; 0.8)	0.00	*0.985*	−0.23 (− 1.28; 0.83)	− 0.04	*0.734*
	*APAD*	12.2 (5.4)												
MM - trunk (kg)	*UC*	23.4 (2.9)	–	–	–	− 0.13 (− 0.37; 0.11)	− 0.09	*0.468*	0.02 (−0.32; 0.37)	0.01	*0.779*	0.06 (−0.4; 0.52)	0.02	*0.689*
	*APAD*	22.9 (2.6)												
FM - legs (kg)^§^	*UC*	8.7 (2.9)	–	–	–	−0.06 (−0.11; − 0.01)	− 0.20	*0.071*	0.02 (−0.05; 0.09)	0.04	*0.555*	0.01 (−0.08; 0.1)	0.02	*0.734*
	*APAD*	8.4 (3)												
MM - legs (kg)	*UC*	16.5 (1.9)	–	–	–	0.00 (− 0.21; 0.21)	0.00	*0.743*	**0.30 (0.00; 0.59)**	**0.17**	***0.038***	**0.40 (0.01; 0.79)**	**0.17**	***0.032***
	*APAD*	16 (1.7)												

*Abbreviations*: *AMD* Adjusted Mean Differences, *EF* Effect Size of difference between groups, *CI* Confidence Interval, *TAP* Test of Attentional Performance, *APAD* Adapted Physical Activity and Diet counseling intervention, *UC* Usual Care, *FM* Fat Mass, *MM* Muscle mass

NOTE: Bold font indicates significant difference. T1 columns contain empty cells because muscular, body composition and attentional outcomes were not measured at T1, except weight and BMI

^*^Baseline value, surgery type and age-adjusted mean differences and their associated 95% confidence interval estimated with linear mixed model

^†^*P*-value for time*arm interaction estimated with linear mixed model

^‡^Log transformed variables: Sit-and-stand 30s, Flexibility: errors – TAP, Working memory: errors and omissions – TAP

^§^Square root transformed variables: Muscle to fat mass ratio, Fat mass - legs only

**Table 4:** Mean values at baseline, and between-group differences and at the end of chemotherapy, the end of radiotherapy and at 6- and 12-month follow-up for behavioral outcomes

		T0 Baseline	T1–18 weeks Mid-intervention			T2–26 weeks End of intervention			T3–52 weeks 6-month follow-up			T4–78 weeks 1-year follow-up		
		*Mean (SD)*	AMD (95% CI)*	*EF*	*P*†	AMD (95% CI)*	*EF*	*P*†	AMD (95% CI)*	*EF*	*P*†	AMD (95% CI)*	*EF*	*P*†
Declared physical activity in MET.min/semaine (GPAQ)														
Total (MET.min/wk) ^¶^	*UC*	1671 (1788)	5.82 (−0.04; 11.67)	*0.16*	*0.108*	4.31 (−1.62; 10.23)	*0.12*	*0.219*	3.92 (−2.7; 10.55)	*0.10*	*0.283*	−1.05 (−8.51; 6.41)	*−0.02*	*0.936*
	*APAD*	1506 (1617)												
Moderate intensity (MET.min/wk)	*UC*	1659 (1791)	293.7 (−140.2; 727.6)	*0.11*	*0.179*	220.8 (−219.5; 661)	*0.08*	*0.273*	418.1 (−87.9; 924.2)	*0.14*	*0.110*	105.4 (− 489.2; 700)	*0.03*	*0.550*
	*APAD*	1428 (1427)												
Leisure (MET.min/wk)	*UC*	663 (1214)	**8.77 (3.87; 13.66)**	***0.29***	***0.001***	**9.61 (4.66; 14.56)**	***0.32***	***0.000***	3.89 (−1.62; 9.39)	*0.12*	*0.071*	2.29 (−3.88; 8.46)	*0.06*	*0.203*
	*APAD*	343 (559)												
Sedentary time (min/day)	*UC*	431 (178)	28.8 (−18.9; 76.6)	*0.10*	*0.311*	−28.1 (−78.0; 21.4)	*−0.09*	*0.450*	−31.4 (−91.2; 28.3)	*−0.09*	*0.440*	−55.0 (− 126.2; 16.3)	*−0.13*	*0.205*
	*APAD*	415 (143)												
Achieved physical activity for an average day (ArmBand)														
Total (min) ^‡^	*UC*	88 (79)				0.24 (−0.11; 0.59)	*0.11*	*0.166*						
	*APAD*	84 (62)												
Moderate intensity (min)	*UC*	86 (76)				14.6 (−11.6; 40.9)	*0.09*	*0.094*						
	*APAD*	83 (61)												
Average intensity (MET.h)	*UC*	2 (0)				0.05 (−0.08; 0.18)	*0.06*	*0.279*						
	*APAD*	2 (0)												
Sedentary time (min)	*UC*	776 (211)				13.3 (−50.9; 77.5)	*0.03*	*0.432*						
	*APAD*	755 (162)												
Nutrient intake per day (3-day record)														
Total energy (kcal) ^‡^	*UC*	1418.2 (414.1)				−0.03 (−0.11; 0.05)	*−0.07*	*0.248*				−0.03 (− 0.23; 0.16)	*−0.03*	*0.600*
	*APAD*	1538.6 (427.4)												
Proteins (g)	*UC*	64.1 (20.2)				3.89 (−0.85; 8.63)	*0.13*	*0.367*				2.2 (−7.09; 11.5)	*0.04*	*0.830*
	*APAD*	66.7 (17.4)												
Animal proteins (g)	*UC*	35.4 (19.6)				3.35 (−1.69; 8.39)	*0.11*	*0.366*				8.92 (−5.49; 23.33)	*0.10*	*0.274*
	*APAD*	36.9 (17.4)												
Vegetal proteins (g)	*UC*	12.8 (7.7)				−0.03 (−1.92; 1.85)	*0.00*	*0.885*				−1.45 (−6.28; 3.39)	*−0.05*	*0.539*
	*APAD*	13.3 (6.9)												
Lipids (g)	*UC*	60 (20.6)				−4.9 (−10.3; 0.54)	*−0.15*	*0.091*				−11.2 (−23.6; 1.1)	*− 0.15*	*0.066*
	*APAD*	64.2 (22.5)												
Saturated fat (g) ^‡^	*UC*	21.9 (7.8)				−0.02 (−0.13; 0.09)	*−0.03*	*0.517*				−0.05 (− 0.34; 0.24)	*−0.03*	*0.648*
	*APAD*	24.8 (9.8)												
Monounsaturated fat (g)	*UC*	22.7 (8.3)				−2.27 (−4.61; 0.07)	*−0.16*	*0.081*				−4.28 (−9.32; 0.75)	*− 0.14*	*0.089*
	*APAD*	24.1 (9.6)												
Polyunsaturated fat (g) ^‡^	*UC*	7.8 (7.2)				−0.07 (−0.21; 0.07)	*−0.08*	*0.408*				−0.05 (− 0.35; 0.25)	*−0.03*	*0.748*
	*APAD*	7 (3.2)												
Total carbohydrates (g)	*UC*	145.4 (51.6)				−4.17 (−17.09; 8.76)	*−0.05*	*0.365*				9.01 (−23.57; 41.59)	*0.05*	*0.692*
	*APAD*	160.6 (52.6)												
Fibres (g)	*UC*	15.2 (5.8)				−0.43 (−2.07; 1.2)	*−0.04*	*0.708*				0.35 (−3.58; 4.28)	*0.01*	*0.834*
	*APAD*	14.7 (5.7)												

*Abbreviations*: *AMD* Adjusted Mean Differences, *CI* Confidence Interval, *EF* Effect Size, *GPAQ* Global Physical Activity Questionnaire, *APAD* Adapted Physical Activity and Diet counseling intervention, *UC* Usual Care

NOTE: Bold font indicates significant difference

*Baseline value, surgery type and age-adjusted mean differences and their associated 95% confidence interval estimated with linear mixed model

^†^*P*-value for time*arm interaction estimated with linear mixed model

^‡^Log transformed variables: Total physical activity – ArmBand, Total energy – 3-day record, Saturated fat – 3-day record, Polyunsaturated fat – 3-day record, Linoleic acid – 3-day record, Linolenic acid – 3-day record

^¶^Square root transformed variables: Total physical activity – GPAQ, Leisure physical activity – GPAQ

### Fatigue

All dimensions of fatigue were significantly decreased in APAD vs UC at T1, with an effect size (ES) of − 0.28 for general fatigue (Table 2). The difference persisted at T2 for all fatigue scores, although significance was not reached for general fatigue (*P* < 0.10). At T3, mental fatigue and reduced activities were significantly reduced. At T4, general fatigue was significantly decreased (ES, − 0.16) as well as physical fatigue (ES, − 0.18) and reduced activities (ES, − 0.17) (Fig. 2).

### QoL, and psychological distress

Global QoL was significantly increased in APAD vs UC at T1, T2, T3, and T4. At T4, significant increases were seen for global QoL (ES, 0.26), physical function (ES, 0.25), role function (ES, 0.18), and social function (ES, 0.23). Anxiety and depression were both significantly reduced at T1 and T2 (ES, − 0.24 and − 0.38 at T2, respectively), but no between-group difference was found afterward.

### Physical, attentional, and anthropometry measures

Lower limb muscle endurance (e.g.*,* sit-and-stand ratio) was significantly increased in APAD vs UC at T2 (ES, 0.22) (Fig. 3). However, there was no evidence of power or force increase (Table 3). An increase in cognitive flexibility (index and number of errors) was observed at T2, but the alert phase index and working memory were not improved. BMI was significantly reduced by − 0.46 kg/m^2^ (ES, − 0.21) in APAD vs UC at T2, and weight was decreased by − 1.19 kg (ES, − 0.21), with borderline significance (*P* = 0.051). Total fat mass was significantly decreased by − 1.10% (ES, − 0.21) at T2. No between-group difference was seen for total muscle mass. Muscle mass from the legs was significantly increased at T3 and T4 (ES, 0.17). Between-group differences regarding muscle endurance, cognitive flexibility, BMI, and fat mass did not persist at the T3 and T4 follow-up points.

### Physical activity and dietary intake

Based on the GPAQ, declared leisure physical activity was significantly increased at T1 (ES, 0.29) and T2 (ES, 0.32), but not afterward (Table 4). No between-group difference was observed in total physical activity and sedentary. Based on accelerometer valid measurements at T0 (*n* = 106) and T2 (*n* = 86) moderate intensity physical activity was not significantly increased at T2 (*P* = 0.094). No significant effect of the intervention was observed on energy and major macronutrient intake at T2 and T4.

### Chemotherapy completion rates

On average, the RDI was 96.0% in UC and 96.7% in APAD (*P* = 0.39). The percentage of participants who received more than 95% of their planned RDI was 65.1% in UC and 78.8% in APAD (Chi-squared, *P* = 0.083).

### Sensitivity analyses

The drop-out rate was significantly different in APAD vs UC at T4 (7% vs 21%, respectively; Chi-squared, *P* = 0.001). There was no significant difference in baseline characteristics in the included patients with complete data vs incomplete data (*P* > 0.05) (Additional file 6: Table S3).

## Discussion

The results of the APAD1 study support that an exercise and diet intervention relieves cancer-related fatigue and QoL during chemotherapy and radiotherapy, with sustainable effects seen at the 1-year follow-up post-intervention, in patients with early BCa. Consistent with our hypotheses, salutary effects were also found on anxiety, depression, leisure physical activity, BMI, fat mass, muscle endurance (sit-and-stand test) and cognitive flexibility at the end of radiotherapy. Although these effects were not maintained at all follow-up times, lean body mass in the legs was significantly increased at the 1-year follow-up. Contrary to our hypotheses, there was no significant effect of the intervention on nutritional intakes, objective physical activity (as measured by accelerometer), working memory, alertness, and completion rates of chemotherapy.

This is the first study to demonstrate that a multicomponent exercise-diet intervention delivered during BCa chemotherapy and radiotherapy had significant benefits on fatigue that are sustainable 1 year after the end of the intervention. According to our knowledge, two previous studies have investigated the effects of a combined diet and exercise intervention during adjuvant therapy for BCa [33, 34]. However, they have not assessed fatigue. In studies that investigated the effect of exercise intervention only during adjuvant therapy for BCa, numerous have reported beneficial effects on fatigue immediately after the intervention [23]; but these trials presented follow-up periods that did not exceed 6 months, and did not include a diet interventional component. These studies reported mixed findings about fatigue at follow-up times [35–40].

Another original finding is the sustainable 1-year post-intervention beneficial effect of the APAD intervention on QoL. Indeed, previous studies testing a combined diet and exercise intervention during adjuvant therapy for BCa [33, 34] did not found any beneficial effects on QoL. In addition, exercise only intervention studies reported mixed findings about QoL at follow-up times (6-month follow-up maximum) [35–40].

Cancer-related fatigue was significantly improved in the APAD vs the UC group at the end of chemotherapy and the end of radiotherapy. The effect of the APAD intervention on general fatigue (ES = − 0.28) at the end of chemotherapy was, as a matter of size, comparable to the average effect size observed on fatigue in a recent meta-analysis that included 22 pooled exercise interventions during adjuvant therapy for BCa (ES = − 0,28) [23].

Other PROs i.e., QoL, anxiety and depression, yielded significant positive results at the end of chemotherapy and at the end of radiotherapy, with small to moderate effect sizes. Regarding QoL, the effect size of the APAD intervention at the end of chemotherapy or radiotherapy (ES = 0.24) was higher than the average effect size observed on QoL in the 13 pooled exercise interventions during adjuvant therapy for BCa (ES = 0,12) [23]. The APAD impact on depression (ES of − 0.38 at the end of radiotherapy) was higher as well than in the 6 pooled exercise studies that measured depression (ES = − 0.15) [23]. In addition, the impact of the APAD intervention persisted with small but significant positive on fatigue and QoL results 1 year after the end of radiotherapy. Post-diagnosis deteriorations in fatigue, QoL and psychological distress have generally been associated with poorer BCa survival in BCa patients [16, 17], making the long-term efficacy (i.e.*,* 1 year follow-up) of the APAD intervention particularly relevant in clinical practice.

Previous combined diet and exercise interventions delivered during chemotherapy [33, 34] did not report any benefits on body fat (%) and BMI. The APAD intervention was efficient to decrease body fat (%) and BMI at the end of intervention. This is a particularly relevant effect in the clinical context of BCa as BMI before and after BCa diagnosis and weight gain after diagnosis have recently been associated with increased mortality in meta-analyses [18, 19].

Mixed findings were observed in the APAD study about muscular outcomes: muscular endurance (as measure by the 30s sit-and-stand test) was improved at the end of the intervention whereas lower limb force or power did not change. Previous combined diet and exercise interventions did not measure these outcomes [33, 34]. However, a recent meta-analysis of the studies with exercise only interventions in BCa receiving adjuvant therapy has reported a pooled significant improvement on strength [23]. Although individual studies effects were inconsistent, it seems that high doses training or important focus on resistance training led to better effects on strength [36, 40, 68].

The APAD intervention yielded improvements on cognitive flexibility at the end of the intervention. To our knowledge, two previous studies investigated the impact of exercise interventions on cognitive function by using the trail-making test (assessing concentration and cognitive flexibility) in BCa patients receiving adjuvant therapy [69, 70]. Although intervention group improved at post- vs pre-intervention, none of the two studies reported a significant difference in the intervention vs the control group. The longer duration of the APAD intervention (i.e., 24 weeks), as compared to 12 weeks in these two previous studies [69, 70] could explain the greater impact of the APAD intervention on cognitive flexibility.

About behavioral outcomes, previous combined interventions including diet and exercise components delivered during chemotherapy [33, 34] both yielded significant changes in dietary intakes, and one in declared total physical activity in the intervention group (post vs pre-intervention) [34]. The APAD intervention had significant favorable impact on leisure time physical activity at the end of chemotherapy and at the end of radiotherapy, but improvements in total physical activity were not significant. Physical activity done in the framework of the APAD intervention was reported in the leisure category, which explain the enhancement of the physical activity type in our study. Regarding dietary intakes, no significant changes were observed in our study in the APAD vs the UC group. The 3-day record method (and foods to nutrition conversion software) we used generated large standard deviations (see Table 4, baseline values for nutrients) that have possibly impair statistical power to detect a between-group difference. The two abovementioned previous studies [33, 34] that demonstrated dietary changes have analyzed dietary data that were collected by food frequency questionnaires.

Except the long term effects on fatigue and QoL, most of the significant outcomes (i.e., anxiety, depression, fat mass, BMI, muscular endurance, cognitive flexibility, physical activity) in the APAD study were limited to the end of chemotherapy (mid-intervention) or the end of radiotherapy (immediately post-intervention) and were not maintained after the end of intervention. In studies with diet and/or exercise intervention, a few have included follow-up measures (6 months follow-up maximum) [35–40], but most of them did not showed effects after the end of the intervention as well [35, 37, 39, 40], except for some PROs [36, 38] as in the APAD study for fatigue and QoL (measured as PROs). One study reported improvements in the 6-min walk test at 6 months post-intervention [38]. Difficulties in maintaining positive outcomes after the intervention could be related to the cessation of supervision and support for keeping behavior changes. This finding may promote the necessity of setting longer intervention models that could include for instance the APAD “in-treatment” module during the 24 weeks of chemotherapy and radiotherapy, followed by a 6-month internet-based “survivor” module designed to maintain behavior change and support autonomy with limited cost. Several telephone- or internet-based diet-exercise interventions in BCa survivors have been tested and yielded health benefits [71–73] with moderate to good adherence rates (from 41 to 87% of adherent patients) [74].

In the APAD study, adherence was estimated to 67 and 97% of completed planned sessions in the exercise and diet components, respectively. Previous diet-exercise interventions delivered to BCa patients during adjuvant therapy did not report completed exercise sessions but only telephone counselling sessions completed as adherence rates, one was 80% [33] and the other one was 92% [34]. In mixed supervised / non-supervised exercise interventions, adherence rates when reported were in the range of 60–80% [75–77] that is in the order of magnitude of the APAD study exercise adherence rate. In the APAD study, adherence was higher for exercise aerobic sessions (71%) than for resistance exercise sessions (58%). In our clinical experience, patients found generally easier to integrate aerobic exercise (e.g., speed walking) in their daily life rather than resistance exercise. In aerobic and resistance exercise interventions that reported separate aerobic and resistance adherence data, the aerobic adherence rate was systematically superior to the resistance rate [36, 78, 79], as we observed in the APAD study.

The APAD1 study was designed to primarily assess in a pragmatic context the effectiveness of exercise and diet in combination compared to the standard of care that was delivered in France; this is why a UC control was chosen. The main drawback of this design is that it does not allow disentanglement of the independent effects of exercise and dietary components. Another limitation is the differential drop-out rate in the APAD vs UC groups. However, according to sensitivity analyses, there was no indication of a selection bias leading to an overestimation of the intervention effect. Although many outcomes were significantly improved in APAD vs UC over time, and an increase was observed in declared leisure physical activity, the between-group difference was not significant for objectively measured physical activity. The physical activity level of the UC group may have partly diluted the effects of the APAD intervention. Given the number of comparisons we made at each time point for the secondary outcomes without adjustment for multiple testing, we would expect a few false discoveries by chance.

Strengths involve the long follow-up of 1.5 years in total for each patient, with assessments repeated five times; and a wide range of outcomes, including both subjective and objective measures. The intervention was theoretically grounded using multiple components and specifically addressing common barriers present in this highly vulnerable population. It was thought to limit patients’ travel-related costs by mixing supervised and home-based sessions. These sessions have been though and tailored to fit with the comings and goings at the hospital of the patients receiving their BCa adjuvant therapy, making of the APAD model an easily transferable model to other hospitals. Sessions content has been described in this article and the protocol of the study [41]. The adherence rates were 67 and 97% for the exercise and diet components, respectively, proving a well-received intervention with high acceptance in our target population. Results should be therefore easily generalizable at other care centers.

## Conclusions

In patients with early BCa, APAD1 was the first trial to address and demonstrate durable improvements in fatigue and QoL at the 1-year follow-up due to a diet-exercise intervention delivered during chemotherapy and radiotherapy. In addition, a beneficial impact of the APAD intervention was found on BMI, fat mass, muscle endurance, cognitive flexibility, anxiety and depression, and declared physical activity at the end of chemotherapy/radiotherapy, with effect sizes indicating small to moderate associations. Therefore, a mixed hospital- and home-based diet-exercise intervention during chemotherapy and radiotherapy provided relief from several treatment-related side effects to women undergoing adjuvant therapy for BCa. Improvements in fatigue, QoL, BMI, and fat mass outcomes are of particular clinical relevance as they have been associated with long term survival of BCa patients [16–19]. In general, most women were willing to participate in the APAD intervention and provided good adherence rates. The APAD study adds evidence to support the role of diet-exercise behavior interventions in BCa patients, and bring new evidence in the particular period of adjuvant chemotherapy and radiotherapy. Future studies using a 2 × 2 factorial design to test the superiority of a diet-exercise intervention vs exercise alone and diet alone are warranted, as well as trials evaluating a long term exercise-diet intervention integrating during- and after-treatment periods. Cancer care centers should consider integrating diet-exercise supportive care into the management of patients with BCa receiving chemotherapy and/or radiotherapy.

## Additional files

Additional file 1: **Figure S1.** The APAD1 study design. (TIF 95 kb)

Additional file 2: **Table S1.** Timeline and education targets of supervised APAD exercise sessions. (DOCX 18 kb)

Additional file 3: **Table S2.** Timeline and education targets of face-to-face APAD diet counseling sessions. (DOCX 18 kb)

Additional file 4: **Figure S2.** Number of exercise sessions achieved each week by the APAD patients. (TIF 94 kb)

Additional file 5: **Figure S3.** Reasons for not achieving planned exercise sessions reported by the APAD patients. (TIF 90 kb)

Additional file 6: **Table S3.** Baseline characteristics of included breast cancer patients with complete data (i.e., the primary outcome has been collected at all of the assessment times) and incomplete data (i.e., the primary outcome is missing at one (or more than one) of the assessment times). (DOCX 26 kb)

## Data Availability

The datasets generated and analysed during the current study are not publicly available due to the risk of identifying individuals from their age in days at testing but are available from ICM Biometric Unit (Sophie.Gourgou@icm.unicancer.fr) on reasonable request.

## Abbreviations

APAD: Adapted Physical Activity and Diet
BCa: Breast cancer
BMI: Body mass index;
ES: Effect size
GPAQ: Global Physical Activity Questionnaire
ICM: Montpellier Cancer Institute
MFI: Multidimensional Fatigue Inventory
MVPA: Moderate to vigorous physical activity
PRO: Patient-reported outcome;
QoL: Quality-of-life
RCT: Randomized controlled trial
RDI: Relative dose index
SD: Standard deviation
UC: Usual care
