# Supplementary figures and images for: Short- and long-term impact of adapted physical activity and diet counseling during adjuvant breast cancer therapy: the “APAD1” randomized controlled trial
Source: BMC Cancer. 2019 Jul 25;19:737. doi: 10.1186/s12885-019-5896-6 (PMC6659309; doi:10.1186/s12885-019-5896-6)

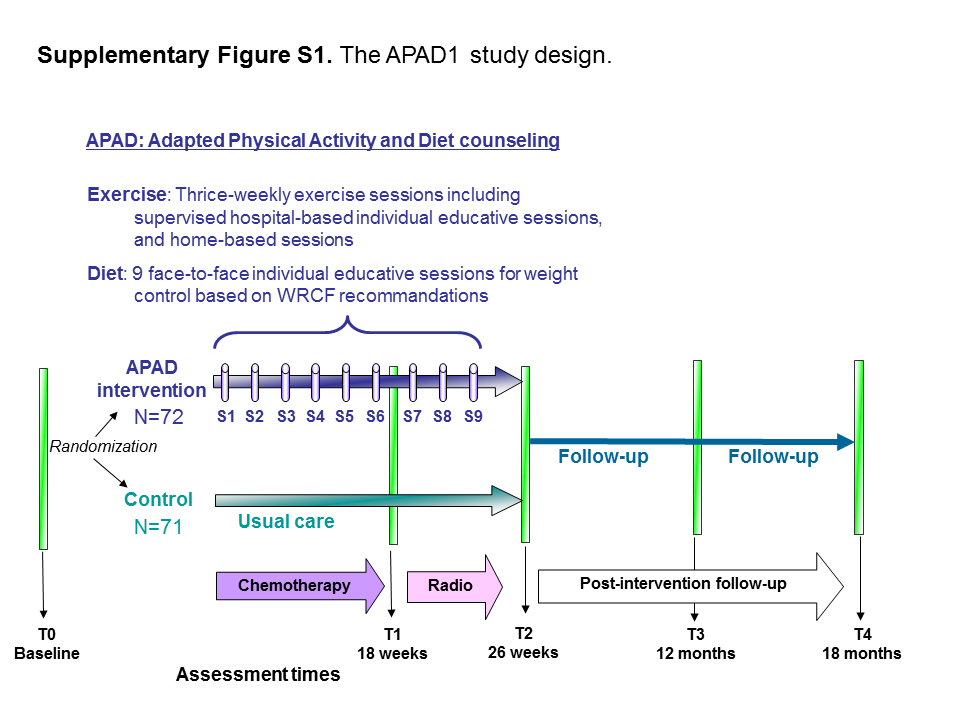

Supplement: Supplementary file 1 — Figure S1. The APAD1 study design. (TIF 95 kb) [file 12885_2019_5896_MOESM1_ESM.tif]

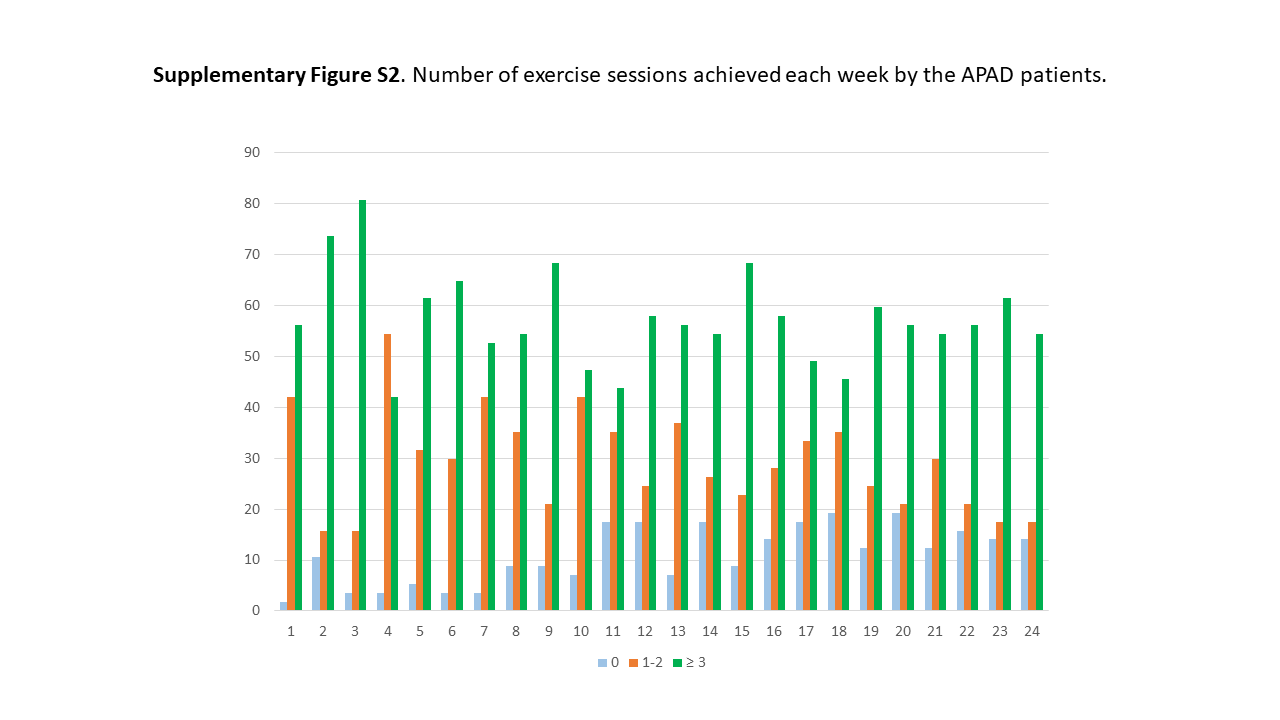

Supplement: Supplementary file 4 — Figure S2. Number of exercise sessions achieved each week by the APAD patients. (TIF 94 kb) [file 12885_2019_5896_MOESM4_ESM.tif]

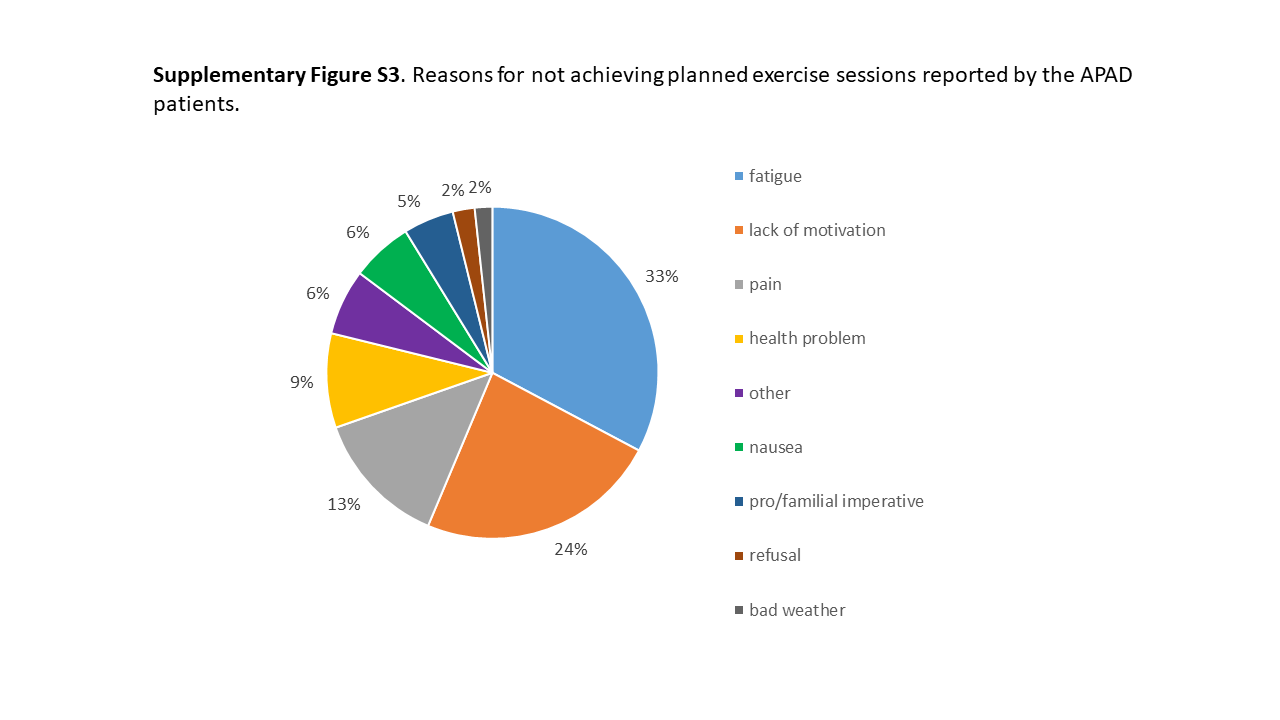

Supplement: Supplementary file 5 — Figure S3. Reasons for not achieving planned exercise sessions reported by the APAD patients. (TIF 90 kb) [file 12885_2019_5896_MOESM5_ESM.tif]
