# Supplementary material for: Short- and long-term impact of adapted physical activity and diet counseling during adjuvant breast cancer therapy: the “APAD1” randomized controlled trial
Source: BMC Cancer. 2019 Jul 25;19:737. doi: 10.1186/s12885-019-5896-6 (PMC6659309; doi:10.1186/s12885-019-5896-6)
Supplement: Supplementary file 2 — Table S1. Timeline and education targets of supervised APAD exercise sessions. (DOCX 18 kb) [file 12885_2019_5896_MOESM2_ESM.docx]

# Table S1: Timeline and education targets of supervised APAD exercise sessions

| **Session no.** | **Treatment time** | **Exercise type** | **Behavior change techniques** | **Description** |
| --- | --- | --- | --- | --- |
| 1 | Chemotherapy 1^st^ cycle  (week 1) | Muscular strength | Provide information about behavior-health link and consequences of practice | Exercise benefits for patients with breast cancer; APAD-Moving workbook presentation; emphasis on strength exercises (information about benefits and muscular groups) |
|  |  |  | Provide instruction and demonstrate the behavior | The exercise specialist describes, shows and realizes strength exercises with the patient |
|  |  |  | Set graded tasks | Number of series/repetitions for each strength exercise |
|  |  |  | Prompt self-monitoring of behavior | Patients are asked to complete activity logs of the APAD-Moving workbook |
|  |  |  | Plan social support or social change | Presentation of “Moving together” forum specially reserved for APAD patients (communication, support, strength exercises videos) |
| 2 | Chemotherapy 2^nd^ cycle  (week 4) | Aerobic exercise | Provide information about behavior-health link and consequences of practice | Aerobic exercise information and benefits for patients with breast cancer |
|  |  |  | Provide instruction and prompt self-control | Heart rate target (50-75% HRmax) in relation with perceived exertion; rating of perceived exertion (Borg scale) |
|  |  |  | Set graded task | Activity type, intensity and duration of home-based aerobic sessions |
|  |  |  | Prompt self-monitoring of behavior | Logs review with APAD-Moving workbook |
|  |  |  | Relapse prevention | Help the patient to manage fatigue in order to avoid failure in maintaining new exercise behavior |
| 3 | Chemotherapy 3^rd^ cycle  (week 7) | Muscular strength | Provide feedback on performance | Summary extraction of accelerometer baseline data; comments, feedback about duration of moderate and vigorous physical activity, sedentary time, steps number |
|  |  |  | Prompt specific goal setting | Behavior goals setting according to general physical activity recommendations |
|  |  |  | Provide instruction and demonstrate the behavior | Emphasis on flexibility (benefits, description and realization of exercises) |
|  |  |  | Prompt self-monitoring of behavior | Logs review with APAD-Moving workbook |
|  |  |  | Plan social support or social change | Forum promotion |
| 4 | Chemotherapy 4^th^ cycle  (week 10) | Aerobic exercise | Prompt barrier identification | Identify barriers to exercising and plan ways of overcoming them |
|  |  |  | Provide contingent rewards | Praise, encouragement |
|  |  |  | Provide information on health-behavior link through therapeutic education | Information about the definition and health benefits of regular physical activity through a quiz game pointing out preconceived ideas |
|  |  |  | Prompt self-monitoring of behavior | Logs review with APAD-Moving workbook |
| 5 | Chemotherapy 5^th^ cycle  (week 13) | Muscular strength | Provide instruction and demonstrate the behavior | Introduction of strength exercises for shoulders and biceps/triceps muscle groups |
|  |  |  | Stress management | Emphasis on relaxation exercises, benefits, abdominal breathing |
|  |  |  | Prompt self-monitoring of behavior | Logs review with APAD-Moving workbook |
| 6 | Chemotherapy  6^th^ cycle  (week 16) | Aerobic exercise | Prompt barrier identification | Identify barriers to exercising and plan ways of overcoming them |
|  |  |  | Time management | Helping the patient to fit the prescribed exercise sessions into a weekly schedule |
|  |  |  | Relapse prevention | Help the patient to manage fatigue in order to avoid failure in maintaining new exercise behavior |
|  |  |  | Prompt self-monitoring of behavior | Logs review with APAD-Moving workbook |
| 7 | Start of radiation therapy  (week 21) | Muscular strength | Provide new instruction and demonstration, set tasks | According to patient’s practice progression, description and realization of higher difficulty level muscle strength exercises |
|  |  |  | Set graded tasks | According to patient’s practice progression, increase of one set of 6 to 12 repetitions per exercise |
|  |  |  | Prompt self-monitoring of behavior | Logs review with APAD-Moving workbook |
| 8 | Mid-radiation therapy  (week 24) | Aerobic exercise | Provide information about behavior-health link | Evidence-based exercise benefits for cancer survivors |
|  |  |  | Plan social support or social change | Presentation of cancer survivors’ associations and sport associations |
|  |  |  | Prompt specific goal setting | Personalized exercise project development for post-treatment period (type of activity, framework, frequency, schedule management) |
|  |  |  | Prompt self-monitoring of behavior | Logs review with APAD-Moving workbook |
| 9 | End of radiation therapy  (week 26) | Muscular strength | Prompt practice | Patients are asked to lead the last supervised exercise session (pro-action and autonomy) |
|  |  |  | Provide general encouragement | Praising or rewarding the patients for their efforts at exercise adherence |
|  |  |  | Agree on behavioral contract | Post-treatment exercise project finalization; provide APAD-Moving brochure recording associations, post-treatment exercise benefits and recommendations, and written personalized exercise project |
