# Supplementary material for: Short- and long-term impact of adapted physical activity and diet counseling during adjuvant breast cancer therapy: the “APAD1” randomized controlled trial
Source: BMC Cancer. 2019 Jul 25;19:737. doi: 10.1186/s12885-019-5896-6 (PMC6659309; doi:10.1186/s12885-019-5896-6)
Supplement: Supplementary file 3 — Table S2. Timeline and education targets of face-to-face APAD diet counseling sessions. (DOCX 18 kb) [file 12885_2019_5896_MOESM3_ESM.docx]

# Table S2: Timeline and education targets of face-to-face APAD diet counseling sessions

| **Session no.** | **Treatment time** | **Behavior change techniques** | **Description** |
| --- | --- | --- | --- |
| 1 | Chemotherapy 1^st^ cycle  (week 1) | Provide information about behavior-health link and consequences | Recommendation-based diet expected benefits for patients with breast cancer; general goals of the diet counseling program |
|  |  | Set graded tasks | Set up a food distribution according to guidelines with the objective of controlling weight during chemotherapy. |
|  |  | Provide therapeutic education | Presentation of detailed well-balanced menus |
|  |  | Prompt self-monitoring of behavior | Patients are asked to complete a 24h-recall the day before the next diet counseling session |
| 2 | Chemotherapy 2^nd^ cycle  (week 4) | Provide instructions (if necessary) | Aiming to adhere to the chemotherapy food distribution in order to control weight according to patient’s BMI, appetite, needs and habits reported in the 24h-recall |
|  |  | Prompt barrier identification | Advice to deal with chemotherapy side effects that could affect dietary intakes (see the “diet counseling” section for details about specific advice) |
|  |  | Provide therapeutic education | Identify nature and specific roles of food groups  Food balance education based on the food pyramid presentation |
|  |  | Prompt self-monitoring of behavior | Patients are asked to complete a 24h-recall the day before the next diet counseling session |
| 3 | Chemotherapy 3^rd^ cycle  (week 7) | Provide instructions (if necessary) | Aiming to adhere to the chemotherapy food distribution in order to control weight according to patient’s BMI, appetite, needs and habits reported in the 24h-recall |
|  |  | Prompt barrier identification | Advice to deal with declared chemotherapy side effects that could affect dietary intakes |
|  |  | Provide information on health-behavior link through therapeutic education | WCRF recommendations based on a quiz game |
|  |  | Prompt self-monitoring of behavior | Patients are asked to complete a 24h-recall the day before the next diet counseling session |
| 4 | Chemotherapy 4^th^ cycle  (week 10) | Provide instructions (if necessary) | Aiming to adhere to the chemotherapy food distribution in order to control weight according to patient’s BMI, appetite, needs and habits reported in the 24h-recall |
|  |  | Prompt barrier identification | Advice to deal with declared chemotherapy side effects that could affect dietary intakes |
|  |  | Prompt self-monitoring of behavior | Patients are asked to complete a 24h-recall the day before the next diet counseling session |
| 5 | Chemotherapy 5^th^ cycle  (week 13) | Provide instructions (if necessary) | Aiming to adhere to the chemotherapy food distribution in order to control weight according to patient’s BMI, appetite, needs and habits reported in the 24h-recall |
|  |  | Prompt barrier identification | Advice to deal with declared chemotherapy side effects that could affect dietary intakes |
|  |  | Provide information on health-behavior link through therapeutic education | Food balance education based on the “APAD fridge game” consisting in the elaboration of 3 balanced meals with the food provided on the picture |
|  |  | Prompt self-monitoring of behavior | Patients are asked to complete a 24h-recall the day before the next diet counseling session |
| 6 | Chemotherapy  6^th^ cycle  (week 16) | Provide instructions (if necessary) | Aiming to adhere to the chemotherapy food distribution in order to control weight according to patient’s BMI, appetite, needs and habits reported in the 24h-recall |
|  |  | Prompt barrier identification | Advice to deal with declared chemotherapy side effects that could affect dietary intakes |
|  |  | Provide information on health-behavior link through therapeutic education | Evidence-based information on the relation of nutrition with cancer through a quiz game pointing out preconceived ideas |
|  |  | Prompt self-monitoring of behavior | Patients are asked to complete a 24h-recall the day before the next diet counseling session |
| 7 | Start of radiation therapy  (week 21) | Set graded tasks | Set up a new caloric distribution according to patient’s BMI aiming to control weight if patient’s BMI<30, or to normalize weight (25≤BMI<30) if patient’s BMI≥30 (see the “diet counseling” section for more details) |
|  |  | Plan social support | Consideration on how other family members can change their behavior to support new patient’s diet goals |
|  |  | Prompt barrier identification | Advice to deal with declared delayed chemotherapy and radiotherapy side effects that could affect dietary intakes |
|  |  | Provide therapeutic education | Teach to read labels and food packaging  Food balance education through an example of complex mixed dish (e.g. lasagna) |
|  |  | Prompt self-monitoring of behavior | Patients are asked to complete a 24h-recall the day before the next diet counseling session |
| 8 | Mid-radiation therapy  (week 24) | Provide instructions (if necessary) | Aiming to adhere to the radiotherapy caloric distribution according to patient’s BMI, appetite, needs and habits reported in the 24h-recall |
|  |  | Prompt barrier identification | Advice to deal with declared delayed chemotherapy and radiotherapy side effects that could affect dietary intakes |
|  |  | Prompt practice through therapeutic education | Food and dishes choice from proposed menus to obtain balanced meals in special contexts such as picnic, fast-food, restaurant |
|  |  | Prompt self-monitoring of behavior | Patients are asked to complete a 24h-recall the day before the next diet counseling session |
| 9 | End of radiation therapy  (week 26) | Provide instructions (if necessary) | Aiming to adhere to the radiotherapy caloric distribution according to patient’s BMI, appetite, needs and habits reported in the 24h-recall |
|  |  | Provide feedback on behavior change | Weight evolution check-up, food and caloric partitioning adherence |
|  |  | Provide general encouragement | Praising or rewarding the patients for their efforts at dietary goals adherence |
|  |  | Prompt specific goal setting | Personalized dietary advice for post-treatment period (food types, caloric partitioning, dietary intakes regularity) |
|  |  | Provide information on health-behavior link and consequences and model the behavior | Post-treatment diet benefits and recommendations, delivery of a booklet summarizing dietary WCRF recommendations |
