# Supplementary material for: Short- and long-term impact of adapted physical activity and diet counseling during adjuvant breast cancer therapy: the “APAD1” randomized controlled trial
Source: BMC Cancer. 2019 Jul 25;19:737. doi: 10.1186/s12885-019-5896-6 (PMC6659309; doi:10.1186/s12885-019-5896-6)
Supplement: Supplementary file 6 — Table S3. Baseline characteristics of included breast cancer patients with complete data (i.e., the primary outcome has been collected at all of the assessment times) and incomplete data (i.e., the primary outcome is missing at one (or more than one) of the assessment times). (DOCX 26 kb) [file 12885_2019_5896_MOESM6_ESM.docx]

# Table S3. Baseline characteristics of included breast cancer patients with complete data (i.e., the primary outcome has been collected at all of the assessment times) and incomplete data (i.e., the primary outcome is missing at one (or more than one) of the assessment times)

|  | **Total**  **(N=143)** | | **Complete**  **(N=108)** | | **Incomplete**  **(N=35)** | | ***P-value*** |
| --- | --- | --- | --- | --- | --- | --- | --- |
|  |  |  |  |  |  |  |  |
| **Sociodemographic and clinical characteristics** | |  |  |  |  |  |  |
| Age, *mean (sd)* | 51.60 | *(10.1)* | 51.41 | *(10.3)* | 52.46 | *(9.7)* | *0.68* |
| Post-menopausal, *N (%)* | 67 | *(46.8)* | 49 | *(45.8)* | 18 | *(54.6)* | *0.43* |
| *Tobacco smoking, N (%)* |  |  |  |  |  |  |  |
| Non-smoker | 65 | *(45.4)* | 52 | *(48.2)* | 13 | *(37.1)* |  |
| Smoker | 36 | *(25.2)* | 26 | *(24.1)* | 10 | *(28.6)* |  |
| Former smoker | 42 | *(29.4)* | 30 | *(27.8)* | 12 | *(34.3)* | *0.54* |
| *Marital status, N (%)* |  |  |  |  |  |  |  |
| Single/divorced/widowed, no child | 3 | *(2.1)* | 1 | *(0.9)* | 2 | *(5.7)* |  |
| Single/divorced/widowed, with child | 15 | *(10.5)* | 10 | *(9.3)* | 5 | *(14.3)* |  |
| Married/Living together, no child | 7 | *(4.9)* | 7 | *(6.5)* | 0 | *(0)* |  |
| Married/Living together, with child | 118 | *(82.5)* | 90 | *(83.3)* | 28 | *(80)* | *0.10* |
| *Education level, N (%)* |  |  |  |  |  |  |  |
| No qualifications | 23 | *(16.1)* | 15 | *(13.9)* | 8 | *(22.9)* |  |
| Secondary level | 29 | *(20.3)* | 18 | *(16.7)* | 11 | *(31.4)* |  |
| Completed high school | 31 | *(21.7)* | 26 | *(24.1)* | 5 | *(14.3)* |  |
| Completed ≥ 2 years at University | 60 | *(42)* | 49 | *(45.4)* | 11 | *(31.4)* | *0.09* |
| *Usual professional status, N (%)* |  |  |  |  |  |  |  |
| Full or part-time employed | 95 | *(66.4)* | 75 | *(69.4)* | 20 | *(57.1)* |  |
| Retired | 36 | *(25.2)* | 26 | *(24.1)* | 10 | *(28.6)* |  |
| Unemployed/Medical leave | 12 | *(8.4)* | 7 | *(6.5)* | 5 | *(14.2)* | *0.26* |
| *Surgery type, N (%)* |  |  |  |  |  |  |  |
| Lumpectomy | 60 | *(42)* | 46 | *(42.6)* | 14 | *(40)* |  |
| Quadrantectomy | 57 | *(39.9)* | 39 | *(36.1)* | 18 | *(51.4)* |  |
| Mastectomy | 26 | *(18.2)* | 23 | *(21.3)* | 3 | *(8.6)* | *0.15* |
| *Cancer stage*, N (%)* |  |  |  |  |  |  |  |
| Stage I | 62 | *(43.3)* | 45 | *(73.8)* | 17 | *(48.6)* |  |
| Stage IIa/IIb | 62 | *(43.3)* | 50 | *(82)* | 12 | *(34.3)* |  |
| Stage IIIa/IIIc | 18 | *(12.6)* | 12 | *(66.7)* | 6 | *(17.1)* | *0.33* |
|  |  |  |  |  |  |  |  |
| **PROs levels at baseline** |  |  |  |  |  |  |  |
| General fatigue, *mean (se)* | 9.52 | *(0.3)* | 9.48 | *(0.35)* | 9.65 | *(0.59)* | *0.81* |
| Physical fatigue, *mean (se)* | 9.36 | *(0.35)* | 9.58 | *(0.41)* | 8.65 | *(0.67)* | *0.28* |
| Mental fatigue, *mean (se)* | 7.86 | *(0.3)* | 7.84 | *(0.34)* | 7.91 | *(0.63)* | *0.98* |
| Reduced activities, *mean (se)* | 9.06 | *(0.32)* | 9.15 | *(0.38)* | 8.76 | *(0.64)* | *0.63* |
| Reduced motivation, *mean (se)* | 6.80 | *(0.26)* | 6.93 | *(0.3)* | 6.38 | *(0.49)* | *0.36* |
| Global QoL, *mean (se)* | 68.19 | *(1.54)* | 67.52 | *(1.72)* | 70.34 | *(3.43)* | *0.44* |
| Physical function, *mean (se)* | 87.61 | *(1.05)* | 87.60 | *(1.21)* | 87.65 | *(2.18)* | *0.91* |
| Role function, *mean (se)* | 83.69 | *(1.77)* | 82.72 | *(1.91)* | 86.87 | *(4.28)* | *0.05* |
| Emotional function, *mean (se)* | 66.43 | *(1.71)* | 67.21 | *(1.8)* | 63.97 | *(4.28)* | *0.69* |
| Cognitive function, *mean (se)* | 85.70 | *(1.53)* | 86.76 | *(1.65)* | 82.35 | *(3.65)* | *0.37* |
| Social function, *mean (se)* | 83.45 | *(1.92)* | 83.80 | *(2.06)* | 82.35 | *(4.66)* | *0.74* |
| Anxiety, *mean (se)* | 11.58 | *(0.27)* | 11.50 | *(0.31)* | 11.82 | *(0.55)* | *0.61* |
| Depression, *mean (se)* | 9.89 | *(0.28)* | 9.87 | *(0.32)* | 9.94 | *(0.56)* | *0.91* |
|  |  |  |  |  |  |  |  |
| **Muscular assessment and body size and composition at baseline** | | | |  |  |  |  |
| Sit-and-stand ratio, *mean (se)* | 0.93 | *(0.01)* | 0.93 | *(0.01)* | 0.94 | *(0.02)* | *0.66* |
| Puissance ratio, *mean (se)* | 0.95 | *(0.01)* | 0.95 | *(0.02)* | 0.95 | *(0.02)* | *0.84* |
| Force ratio, *mean (se)* | 1.00 | *(0.01)* | 1.00 | *(0.02)* | 0.99 | *(0.02)* | *0.73* |
| BMI, *mean (se)* | 25.50 | *(-5.3)* | 25.32 | *(0.5)* | 26.25 | *(0.9)* | *0.36* |
| Obese, *N (%)* | 26 | *(-18.2)* | 17 | *(15.9)* | 9 | *(25.7)* | *0.24* |
| Muscle Mass to Fat Mass ratio, *mean (se)* | 2.22 | *(0.08)* | 2.24 | *(0.08)* | 2.16 | *(0.18)* | *0.25* |
|  |  |  |  |  |  |  |  |
| **Behavioral outcomes at baseline** |  |  |  |  |  |  |  |
| Total declared physical activity in (GPAQ) (MET.min/wk), *mean (se)* | 1587.32 | *(142.64)* | 1454.26 | *(144.3)* | 2010.00 | *(376.17)* | *0.35* |
| Total physical activity for an average day (ArmBand) (min/day), *mean (se)* | 85.75 | *(6.83)* | 85.81 | *(7.45)* | 85.48 | *(17.1)* | *0.62* |
| Total energy intake (kcal), *mean (se)* | 1481.52 | *(36.22)* | 1512.11 | *(41.75)* | 1376.93 | *(70.34)* | *0.12* |
